# Full Genome of *Phialocephala scopiformis* DAOMC 229536, a Fungal Endophyte of Spruce Producing the Potent Anti-Insectan Compound Rugulosin

**DOI:** 10.1128/genomeA.01768-15

**Published:** 2016-02-25

**Authors:** Allison K. Walker, Samantha L. Frasz, Keith A. Seifert, J. David Miller, Stephen J. Mondo, Kurt LaButti, Anna Lipzen, Rhyan B. Dockter, Megan C. Kennedy, Igor V. Grigoriev, Joseph W. Spatafora

**Affiliations:** a Department of Chemistry, Carleton University, Ottawa, Ontario, Canada; b Agriculture and Agri-Food Canada, Ottawa, Ontario, Canada; c U.S. Department of Energy Joint Genome Institute, Walnut Creek, California, USA; d Department of Botany and Plant Pathology, Oregon State University, Corvallis, Oregon, USA

## Abstract

We present the full genome of *Phialocephala scopiformis* DAOMC 229536 (Helotiales, Ascomycota), a foliar endophyte of white spruce from eastern Quebec. DAOMC 229536 produces the anti-insectan compound rugulosin, which inhibits a devastating forestry pest, the spruce budworm. This genome will enable fungal genotyping and host-endophyte evolutionary genomics in inoculated trees.

## GENOME ANNOUNCEMENT

*Phialocephala scopiformis* (Helotiales, Ascomycota) strain DAOMC 229536 was isolated as a foliar endophyte of white spruce (*Picea glauca*) from eastern Quebec. It produces the anti-insectan secondary metabolite rugulosin (1), which inhibits feeding by spruce budworm, a devastating commercial forestry pest (2). Budworm damage results in profound habitat disturbance, reduces sustainable wood supply, and affects the carbon balance of the boreal forest. Inoculated trees are more tolerant of the spruce budworm both in nurseries and under field conditions. The growth rate of the insect pest is reduced, exposing larvae to increased disease and predation by birds and reducing the damage to trees (3–5). In recent years, millions of conifer seedlings in New Brunswick production nurseries were inoculated with this strain to reduce the need for spraying chemical insecticides (6), and beneficial effects have persisted more than a decade postinoculation (7). The genome sequence of *P. scopiformis* will enable studies of fungal genome evolution, fungal genotyping, and host-endophyte evolutionary genomics in the inoculated forests.

The *P. scopiformis* genome was sequenced using the Illumina platform. Genomic DNA was extracted using a modified Joint Genome Institute (JGI) cetyltrimethylammonium bromide (CTAB) and genomic tip (Qiagen) protocol; total RNA was extracted using a modified Trizol method. DNA was sheared and size selected to produce two 300 bp insert size fragments and 4 kb Cre-lox long-mate-pair libraries, and sequenced on Illumina HiSeq in 2 × 150 bp and 2 × 100 bp reads, respectively. Stranded cDNA libraries were generated using the Illumina TruSeq stranded RNA LT kit from mRNA fragmented and reverse transcribed using random hexamers and SSII (Invitrogen) followed by second strand synthesis. The fragmented cDNA was treated with end-pair, A-tailing, adapter ligation, and 10 cycles of PCR, and sequenced on HiSeq in 2 × 150 bp reads format.

Illumina reads of stranded RNA-seq data were assembled into consensus sequences using Rnnotator (v2.5.6 or later) (8). Genomic data were filtered and assembled with AllPathsLG version R49403 (9) to produce a 48,876,257 bp assembly in 71 scaffolds (*N*_50_ = 1.3 Mb) and 302 contigs (*N*_50_ = 433.5 Kb). Mapped to the genome assembly were 99.42% of assembled consensus RNA sequences. A mitochondrion was assembled separately with AllPathsLG into a single 35.9 kb contig.

The genome of *P. scopiformis* was annotated using the JGI Annotation Pipeline (10) to identify 18,573 genes, over 80% of which are supported by the transcriptome. Genome assemblies and annotation are available via the JGI fungal portal MycoCosm (10).

### Nucleotide sequence accession number.

The genome of *Phialocephala scopiformis* DAOMC 229536 has been deposited in GenBank under the accession number LKNI00000000.
