# Erratum for Walker et al., Full Genome of *Phialocephala scopiformis* DAOMC 229536, a Fungal Endophyte of Spruce Producing the Potent Anti-Insectan Compound Rugulosin

**DOI:** 10.1128/genomeA.00510-16

**Published:** 2016-05-26

**Authors:** Allison K. Walker, Samantha L. Frasz, Keith A. Seifert, J. David Miller, Stephen J. Mondo, Kurt LaButti, Anna Lipzen, Rhyan B. Dockter, Megan C. Kennedy, Igor V. Grigoriev, Joseph W. Spatafora

**Affiliations:** a Department of Chemistry, Carleton University, Ottawa, Ontario, Canada; b Agriculture and Agri-Food Canada, Ottawa, Ontario, Canada; c U.S. Department of Energy Joint Genome Institute, Walnut Creek, California, USA; d Department of Botany and Plant Pathology, Oregon State University, Corvallis, Oregon, USA

## ERRATUM

Volume 4, no. 1, e01768-15, 2016. Page 2: The final sentence in the Funding Information should read as follows. “This work was funded by U.S. Department of Energy (DOE) under grant DE-AC02-05CH11231.”

